# Cardiopulmonary exercise testing provides a predictive tool for early and late outcomes in abdominal aortic aneurysm patients

**DOI:** 10.1308/003588412X13171221591493

**Published:** 2012-07

**Authors:** TY Tang, PD Hayes, JR Boyle

**Affiliations:** Cambridge University Hospitals NHS Foundation Trust,UK

## Abstract

Thompson AR, Peters N, Lovegrove RE *et al*. Cardiopulmonary exercise testing provides a predictive tool for early and late outcomes in abdominal aortic aneurysm patients. *Ann R Coll Surg Engl* 2011; **93**: 474–481.

We read with interest the paper by Thompson *et al* on the use of cardiopulmonary exercise testing (CPET) as a predictive tool for early and medium-term outcomes in patients undergoing open abdominal aortic aneurysm (AAA) repair. Although the authors allude to this, it is important to highlight that despite being prospective in nature, there are no data on causes of death. The group turned down for surgery appears no different in terms of co-morbidities than those offered surgery. The simple explanation for the higher mortality in the patients deemed unfit for surgery may have been simply AAA rupture. There were only 14 deaths – would it have been useful to find out the specific cause of death from the coroner’s office?

In addition to survival, we agree that it is useful to consider other outcome measures such as length of hospital stay, intensive care (ITU) bed requirement and post-operative cardiorespiratory complications since these are key considerations in deciding whether a patient should undergo major vascular surgery. The 30-day outcome measures of post-operative inotropic requirement and ITU stays are both subjective measures. ITU stay may for example be determined by the day of surgery, more likely to be longer if the operation is in the afternoon or if surgery is planned at the end of the week. The high percentage requiring inotropic support (36%) post-operatively may reflect local anaesthetic practice and use of epidural catheters.

Can the authors justify the use of these subjective variables as a measure of 30-day outcome over traditional endpoints such as myocardial infarction, respiratory and renal failure, and mortality? Why did the authors choose the dated Detsky index (1986) as a measure of physiological scoring? The American Heart Association now advocates the use of the modified Lee score.^1^ Furthermore, none of the scoring tools used in the study were able to predict 30-day major morbidity or mortality. This may have been due to the small operative group as V-POSSUM and APACHE II have been previously shown to predict outcome after open AAA repair in larger groups.^2^

There does not seem to be any ethical approval sought for this study. Do the authors feel it was ethical to offer or turn down patients for open AAA repair on the basis of CPET and in a contemporary study such as this, why were those not offered open surgery not referred to another vascular centre for consideration of EVAR?

One of the key components underlying the framework of improving the results of elective AAA repair in the UK^3^ is pre-operative care and undertaking some form of formal risk assessment to allow correction of any adverse clinical features to reduce the risk of intervention. This study found that CPET’s anaerobic threshold predicted mid-term survival but to use this measure as a basis of turning down a patient for open AAA repair is presumptuous. The evidence for use of CPET in determining those patients fit enough to undergo open AAA repair remains sparse. Carlisle *et al* found that CPET identified patients unlikely to survive in the mid-term, even after successful AAA repair^4^ and a recent publication has showed that AAA patients can undergo CPET safely.^5^ However, how do you optimise a patient’s pre-morbid state based on CPET testing and would it affect what most vascular surgeons or anaesthetists do peri-operatively? Probably not.
